# Supplementary material for: Geometric deep learning of protein–DNA binding specificity
Source: Nat Methods. 2024 Aug 5;21(9):1674–83. doi: 10.1038/s41592-024-02372-w (PMC11399107; doi:10.1038/s41592-024-02372-w)
Supplement: Supplementary file 1 — Supplementary Figs. 1–12, discussion and Table 1. [file 41592_2024_2372_MOESM1_ESM.pdf]

nature methods

Supplementary information https://doi.org/10.1038/s41592-024-02372-w

Geometric deep learning of protein–DNA binding specificity

In the format provided by the authors and unedited

Supplementary Information

Table of Contents

1. Datasets 2
Fig. S1. 3
2. Ungapped local alignment 3
3. Representing DNA 4
Fig. S2. 6
4. Representing protein 6
5. DeepPBS architecture 6
Fig. S3. 7
6. Training, cross-validation, and benchmarking 7
Fig. S4. 8
7. Additional discussion on behavior of metrics 9
8. Additional details associated with application of DeepPBS on predicted structures 9
9. Molecular Dynamics (MD) simulation of Exd-Scr–DNA system 11
10. Additional details on application of DeepPBS to MD simulation of Exd-Scr–DNA system 11
11. Measures ensuring prevention of overfitting to protein sequences 12
12. Details on outliers seen on the benchmark set performance presented in Fig. 2a 12
Fig. S5. 13
Fig. S6. 14
Fig. S7. 15
Fig. S8. 16
Fig. S9. 17
Fig. S10. 18
Fig. S11. 19
Fig. S12. 20
Table S1. 21
Supplementary References 22

1. Datasets

We collected structural data from the Protein Data Bank (PDB)[1] and binding specificity data from JASPAR (version 2022)[2] and HOCOMOCO (v11 core collection)[3]. JASPAR catalogues a comprehensive set of experimental binding specificity data for proteins from different species obtained through various types of experimental platforms. HOCOMOCO consists of mainly chromatin immunoprecipitation followed by sequencing (ChIP-seq)[4] data for human and mouse proteins.

Next, we searched for protein-DNA co-crystal structures available in the PDB (Dec 2022) for each position weight matrix (PWM) available to us using corresponding UniProt IDs. We employed DSSR[5] to check for and annotate the existence of one contiguous double helical region in these structures. In our application, we focused on double-stranded DNA only and discarded structures that did not conform to this requirement. Base modifications were replaced by their parent base identity. A total of 1,155 PDB chain IDs were filtered into the dataset. For each structure (biological assembly containing a chain of interest) in the dataset, a corresponding PWM was paired with it. If a PWM existed in both JASPAR2022[2] and HOCOMOCOv11[3], one was randomly chosen. PWMs were trimmed to remove uninformative terminal regions with a 0.5 information content (IC) threshold. For each structure, we aligned the corresponding PWM to the DNA helix using an ungapped local alignment (Supplementary Section 2), annotating the region on the DNA helix where predictions should be made and the loss computed during training. For source code and further details of data cleaning and pre-processing, see the Data/Code Availability section.

We clustered the protein chains using CD-HITv4.8.1[6] with a 40% sequence similarity threshold for clustering, resulting in 189 clusters. This step ensures that our dataset does not overrepresent any particular protein sequence. Next, we sampled up to five members from each cluster, prioritizing biological assemblies where the chain of interest has more contacts with the DNA region where the PWM was aligned into a fold. Full list of these memberships is available in Extended Data. We set the cutoff for alignment length to be at least five base pairs. We split this set of structures into five folds to create a cross-validation set. A schematic representation of this process is shown in Fig. S1a. Experimental and species diversity of the gathered cross-validation dataset are shown in Fig. S1b.

Structures that were not included in the cross-validation dataset were resampled, selecting up to five per cluster following the same criterion. This resulted in 130 datapoints, which we used as a benchmark set. Predictions on this set were only calculated once, after finalizing all models. The family distribution of this set (Fig. 2c) differs from that of the cross-validation set (Fig. S5b).

The PWM of the same protein differed slightly between JASPAR[2] and HOCOMOCO[3] (example shown in Fig. S1c for human estrogen receptor). This observation indicates that there is an inherent limit on what can be possibly learned, signifying noise in collected knowledge. To quantify the performance limit on the dataset based on this phenomenon, we computed the distribution of performance metrics across all unique PWMs appearing in both databases (111 cases).

Alanine scanning mutagenesis involves measuring changes in binding free energy ($\Delta\Delta G$) when performing the same binding experiment for a given protein, with a specific residue mutated to alanine. We used an already gathered dataset[7] of alanine scanning mutagenesis experiments for protein-DNA structures. We filtered the dataset to make it suitable for our context. Specifically, we removed cases involving single-stranded DNA. Mutations to alanine residues with $\Delta\Delta G$ values within 0–3 kcal/mol were retained. We removed cases in which no heavy atom of the mutated residue was within 5 Å of DNA, because our model only assigns importance scores within this range. The final dataset is summarized in Table S1.

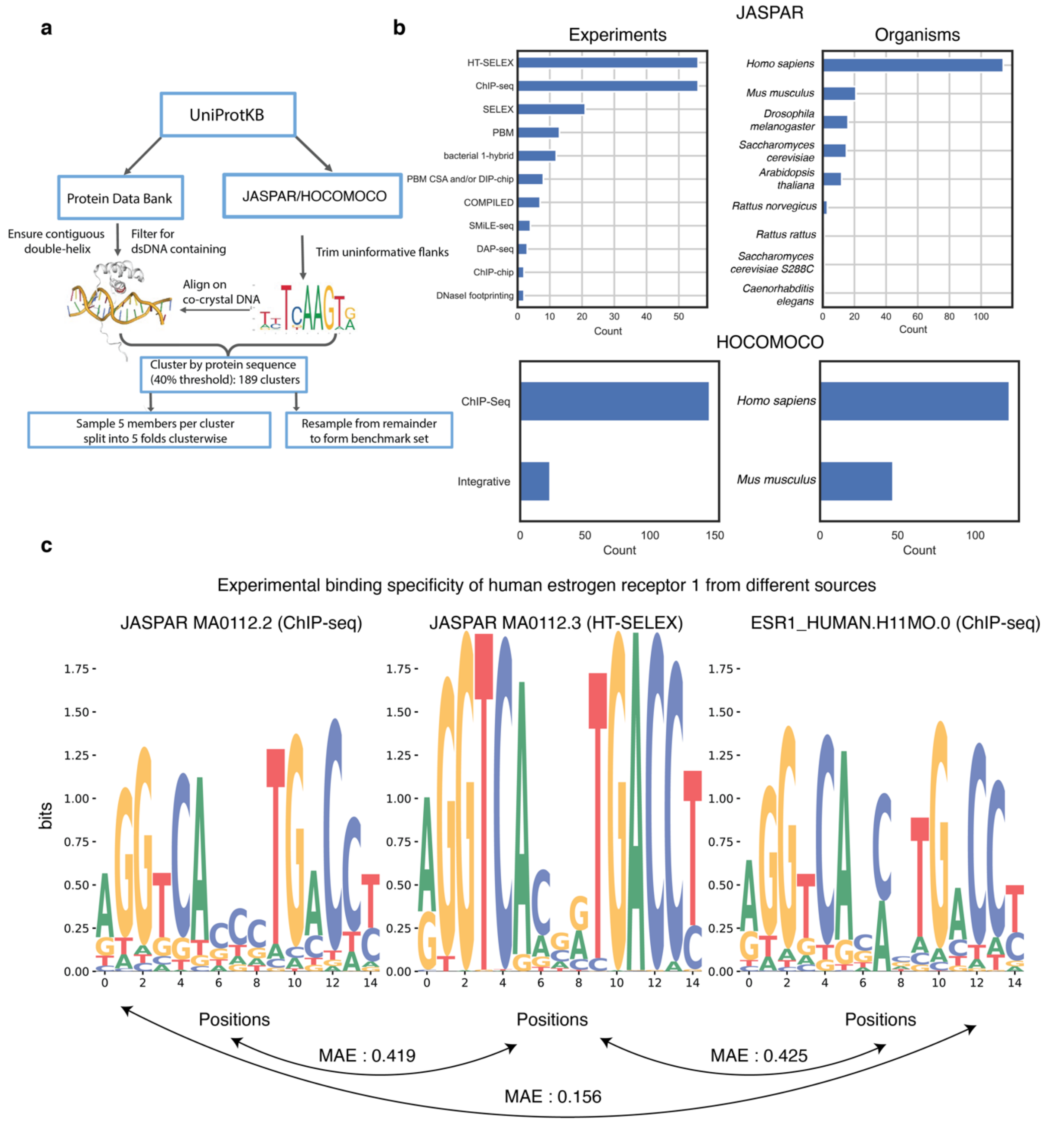

Fig. S1.

Dataset details. (a) Schematic representation of process for combining data sources. **(b)** Distribution of source experiments and species in constructed cross-validation set. **(c)** Example illustration demonstrating differences in binding specificity data for the same protein (human estrogen receptor 1) from different experiments and databases. Mean absolute error (MAE) over columns among three cases.

2. Ungapped local alignment

Alignment of experimental PWMs to the corresponding co-crystal structure derived DNA is an important step for correctly annotating experimental protein-DNA structural data for model training and evaluation. This alignment needs to be ungapped and should prioritize alignment of higher IC columns from the PWM. Hence, we used an IC-weighted Pearson correlation coefficient (PCC) scoring scheme for the alignment, given by:

$$ICWeightedPCC(Col_{PWM}, Col_{DNA}) = PearsonR(Col_{PWM}, Col_{DNA}) \times \frac{1}{2} IC(Col_{PWM})$$

where $PearsonR$ refers to a standard PCC, and IC refers to the information content calculated for a probability simplex with a uniform background, in this context:

$$IC([P_A, P_C, P_G, P_T,]) = \sum_{i \in [A,C,G,T]} \frac{\log(P_i)}{\log(0.25)}$$

<u>Algorithm1 (Pseudocode for ungapped alignment of PWM onto co-crystal DNA using ICWeightedPCC scoring)</u>

```
function ungappedAlign(seq,  pwm):
  // ungapped alignment needs Length X 4 arrays
  max_score ← -9999
  opt_i ←0
  opt_j ←0
  opt_k ←0
  l ← length(seq)
  s ←length(pwm)
  for i from 0 to s-1:
      for k from 0 to s-i:   /// k overlap length
          for j from 0 to l-k:
              score ←0

              for col from 0 to k-1:
                  col_score ←ICWeightedPCC(pwm[i:i+k,:][col,:], seq[j:j+k,:][col,:])
                  score ←score + col_score
              if score > max_score:
                  max_score ←score
                  opt_i ←i //alignment start for PWM
                  opt_j ←j //alignment start for Seq
                  opt_k ←k //alignment length
  return opt_i, opt_j, opt_k, max_score
```

Note, the max_score is length dependent. To compare these values across different protein-DNA structures (as in Fig. 2b, S10b), we have to divide by the overlap length (opt_k).

3. <u>Representing DNA</u>

Our framework must consider several important factors for representing DNA. First, our model observes the structure of DNA in the input, but will predict a one-dimensional (1D) representation (a PWM). Thus, from an engineering perspective, it is beneficial to have the same number of features per base pair. Second, the input co-crystal structure derived DNA has a sequence; depending on the use case, we may or may not want our model to observe this sequence. Moreover, because experimental structural data are sparse, the co-crystal derived sequence has a strong potential for overfitting if observed by the model in the input. Therefore, in general, we want to symmetrize each base pair such that all sequence information is lost, but the global shape of the double helix is preserved.

With these points in mind, we developed a coarse-grain symmetrized representation of DNA, where each base pair is represented by 11 points: two points for the phosphate moiety on each strand, two points for the sugar moiety, four points for the major groove, and three points for the minor groove. Major and minor groove points are placed symmetrically in the base-pair plane, so that they do not possess any particular base identity but roughly correspond to the major and minor groove chemical positions known[8] to be used

for base readout. The phosphate moiety is represented by the coordinate of the phosphorus atom. The sugar moiety is represented by the average coordinate of all sugar heavy atoms. The three minor groove points divide the line segment connecting the two C1' atoms into four equal segments. The base-pair plane is determined by the triangle connecting the two C1' atoms and point O (average of atoms N1 and N9). Next, we move perpendicular (to the minor groove line segment) in this plane from either C1' for 3.75 Å and expand the line segment by another 1.54 Å in either direction. The line segment is divided into five equal segments to determine positions of the four major groove points. Additionally, the central two major groove points are shifted by an additional 1 Å. This geometric construction is based solely on domain knowledge; no learning is employed to estimate any parameter. Fig. S2a shows a schematic representation of this process for an A-T base-pair. The only base atoms used for this process are N1 and N9, making it agnostic of base identity.

Fig. S2b shows an example transformation of a DNA structure to a symmetrized helix (sym-helix) using the described process. Fig. S2c shows one C-G base pair overlayed with sym-helix points computed for the corresponding base pair. As a result, the DNA structure is represented as $G^d = (V^d, X^d, N^d)$. V^d represents coordinates of the sym-helix points, and X^d represents point-level DNA features, which reflect a one-hot encoded annotation of the 11 positions in the symmetrized base-pair representation. If desired, we can reintroduce the DNA sequence ('DeepPBS with DNASeqInfo' model) by including base-pair-specific chemical group features for each point to X^d, as in Chiu et. al. 2023[8]. For each point $v \in X^d$, we also define an interaction vector N_v^d. These vectors act as reference directions in the base-pair frame. They are used to compute relative orientation-based features coupled with vectors N^p on the protein graph (refer to Section 4). For the phosphate point, this vector is the average direction of the two double-bonded oxygens; for the sugar point, this vector is the direction of the C4'-C5' bond. For the seven major and minor groove points, these directions are determined by connecting each point to the centroid of the heptagon formed by these points. Fig. S2e shows the arrangement of these vectors on a sym-helix. These directions do not encode any base-specific information and only serve to inform the relative orientation of a sym-helix point in the context of binding. In addition, we include 14 DNA shape features[9,10,35] denoted as X^s, which are base-pair level features (Fig. S2d). These features are: buckle, shear, stretch, stagger, propeller twist, opening, shift, slide, rise, tilt, roll, helix-twist, major groove width, and minor groove width. 3DNA v2.3[10] and Curves 5.3[9,35] were used to calculate these values. Mean-padding was used to offset inter-base-pair features (shift, slide, rise, tilt, roll, helix-twist).

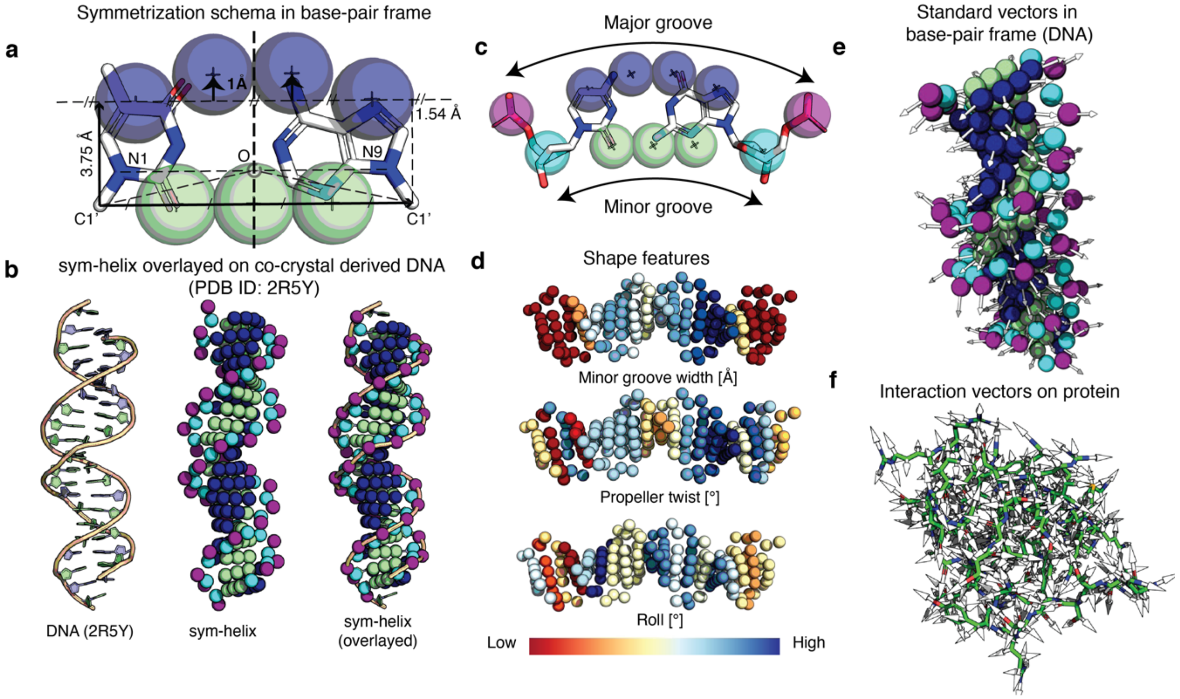

Fig. S2.

Data Representation. (a) Coarse-grain symmetrization schema at DNA base-pair level. **(b)** Example illustration showing how the computed sym-helix compares with the original structure. Each sym-helix point is shown as a sphere (1.5 Å radius) for visibility. **(c)** Symmetrized base-pair representation on one C-G base pair (four major groove points, three minor groove points, and two points each for sugar and phosphate moieties). **(d)** Example computed shape features overlayed on sym-helix as base pair-level features. **(e)** Standard vectors computed on sym-helix, used by the network to correlate orientation information with **(f)** interaction vectors on protein atom-graph, based on average direction of covalent bonds for each heavy atom.

4. Representing protein

In our framework, the protein structure is viewed as a spatial graph $G^p = (V^p, X^p, E^p, N^p)$, where the coordinates of the heavy atoms constitute the vertices V^p. For each vertex $v \in V^p$, we define a set of features X_v^p which include the one-hot encoded atom type, solvent-accessible surface area of the atom, charge, radius, circular variance (7.5 Å radius), and Atchley factors[11]. The edges E^p of the protein graph are determined by the covalent bonds; namely, if vertices u and v have a covalent bond between them, then $(u, v) \in E^p$. The edges are unordered. Lastly, to encode directionality of protein side-chain atoms, we encode a unit vector N_v^p for each vertex v, computed by averaging the directions of covalent bonds associated with each heavy atom (Fig. S2f).

5. DeepPBS architecture

The architecture of DeepPBS is modular. First, the **ProteinEncoder** module applies spatial graph convolutions on the protein graph to aggregate neighborhood environment information for each protein heavy atom. Initially, a fully connected embedding layer is applied to $X_v^p \ \forall\, v \in G^p$, which expands the dimensionality of X_v^p to 10 dimensions. Four layers of crystal graph convolutions (CGConv)[12] are applied. The first two layers use only covalent bond edges, and the next two layers use distance-based edges with a 4Å radius. The mathematical description of the message-passing scheme for CGConv is as follows:

$$X_v^p \leftarrow X_v^p + \left(\frac{1}{|\mathcal{N}(v)|}\right) * \sum_{u \in \mathcal{N}(v)} \sigma\left(z_{uv} W_f + b_f\right) \odot g(z_{uv} W_s + b_s)$$

where $\mathcal{N}(v)$ denotes the neighbors of $v \in V^p$, and $z_{uv} = [X_v^p, X_u^p, e_{uv}]$ denotes the concatenation of target node features, source/neighboring node features, and edge features (here, the distance between u and v). In addition, σ denotes the sigmoid function, and g denotes the softplus function. A Rectified Linear Unit[13] (ReLU) activation function is applied after each round of graph convolutions. This marks the end of the ProteinEncoder module.

The sym-helix point features X_v^d are embedded into a 10-dimensional (10D) space using fully connected neural network layers and are used in the next module, the **Bipartite Geometric Network (BiNet)**. In this module, aggregated information on G^p are pulled onto the sym-helix by performing geometry-aware bipartite convolutions. We use a modified version of point pair feature convolutions (PPFConv)[14], which we call a bipartite ResidualPPFConv. The message-passing update scheme associated with it is as follows:

$$X_v^d \leftarrow X_v^d + \gamma_\theta\left(\sum_{u \in \mathcal{N}(v), u \in V^p} h_\theta\left(X_u^p, \left||d_{uv}|\right|, \angle(N_v^d, d_{uv}), \angle\left(N_u^p, d_{uv}\right), \angle\left(N_v^d, N_u^p\right)\right)\right)$$

where d_{uv} denotes the line segments connecting a sym-helix point v and a protein heavy atom point u. h_θ is a transformation parametrized by fully connected neural networks. We set γ_θ to be the identity transformation. Four separate ResidualPPFConvs are applied for the major groove, minor groove, phosphate, and sugar points, respectively (from neighbors within 5 Å), followed by ReLU activation. At this stage, we have aggregated all local chemical and geometric interaction contexts onto the sym-helix.

The final module is the **CNN-Predictor** module. We flatten the helix into a 1D base pair-level representation and apply a Multi Layered Perceptron (MLP) to reduce the dimensionality for each base-pair to 32. We concatenate precomputed helix shape features to aggregated base pair-level features. This step allows the network to make connections/correlate patterns between the aggregated information from BiNet and the global shape of the helix. We apply two rounds of 1D convolutions of filter size 3 with a stride of 1. We also apply relevant padding and set output feature size of 8, followed by ReLU activation, making the effective field of view five base-pairs. Next, we apply an MLP for each base-pair to generate logits for predicted base probabilities ($[L_A, L_C, L_G, L_T]$) for corresponding base-pairs. We apply a SoftMax[15] activation to generate the output DNA base probabilities ($[P_A, P_C, P_G, P_T]$). A global temperature parameter (T_{glob}) is learned for SoftMax through the training process. Fig. S3 schematically describes the DeepPBS architecture.

$$P_i = \frac{e^{\frac{L_i}{T_{glob}}}}{\sum_{j \in \{A,C,G,T\}} e^{\frac{L_j}{T_{glob}}}} \; \forall \, i \in \{A, C, G, T\}$$

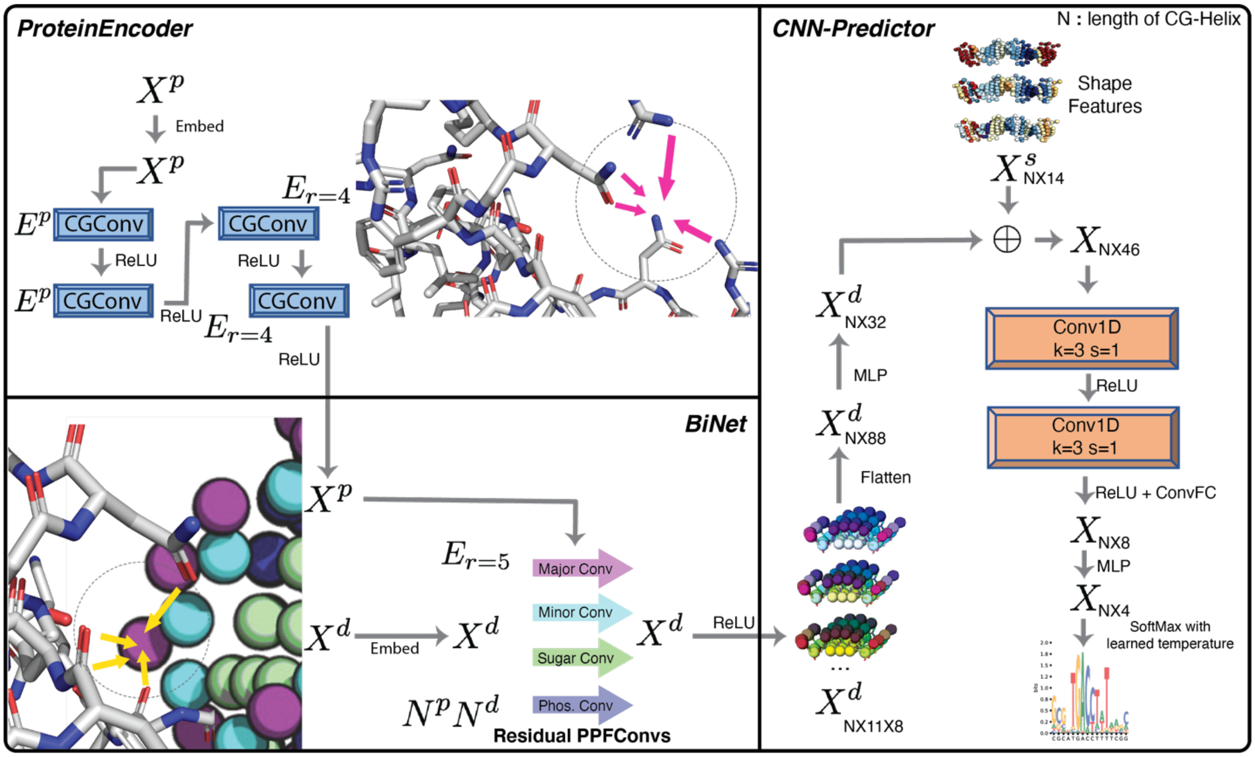

Fig. S3.

DeepPBS architecture. The DeepPBS architecture can be compartmentalized into three modules: ProteinEncoder, which encodes the protein neighborhood through spatial graph convolutions; BiNet, which consists of a network of bipartite geometric convolutions from the protein graph (same notation as in Supplementary Section 4, $G^p = (V^p, X^p, E^p, N^p)$) to sym-helix points (DNA) (same notation as in Supplementary Section 3, $G^d = (V^d, X^d, N^d)$); and CNN-Predictor, which flattens the aggregated sym-helix features into a 1D representation, adds shape features (X^s), and applies 1D convolutional layers followed by fully connected prediction layers. The final logits are converted into probability using a SoftMax activation with a learned temperature parameter. $E_{r=x}$ represents edges determined by vertices/points within a specified radius of length x. Each sym-helix point is shown as a sphere (1.5 Å radius) for visibility.

6. Training, cross-validation, and benchmarking

Five models were trained for each of the four types: *DeepPBS*, *DeepPBS GrooveReadout*, *DeepPBS ShapeReadout*, and *DeepPBS with DNA SeqInfo*. Each model was trained on four folds of the

constructed cross-validation set. Training was conducted for 50 epochs with early stopping on an NVIDIA RTX A4000 using an Adam[16] optimizer, with a learning rate of 0.001 and weight decay of 0.0001. Hyperparameters were selected based on domain knowledge and training curves. For every datapoint, two forward passes were made to account for reverse compliment predictions for both strand directions (with relevant index transformations for input; refer to Data/Code Availability). Outputs were concatenated, and MAE loss was calculated with ground truth (corresponding PWM and its reverse compliment concatenated). Predictions were made on the corresponding fifth/validation fold with each model to gather predictions for all datapoints in the 5-fold dataset. These predictions were used to report metrics in Fig. S5a-c.

For benchmarking purposes, ensemble averaged (of the five trained cross-validation models) predictions are used Fig. 2a-d. The ensemble is also used for results presented in Fig. 3, 4, 5, and Fig. S5b,d, S6, S7, S8, and S9.

All core DeepPBS code was written in python3.9+ with various pythonic dependencies (full list available at GitHub). Packages used for geometric deep learning are pytorch1.12+ and torch-geometric (pyg v2.0+).

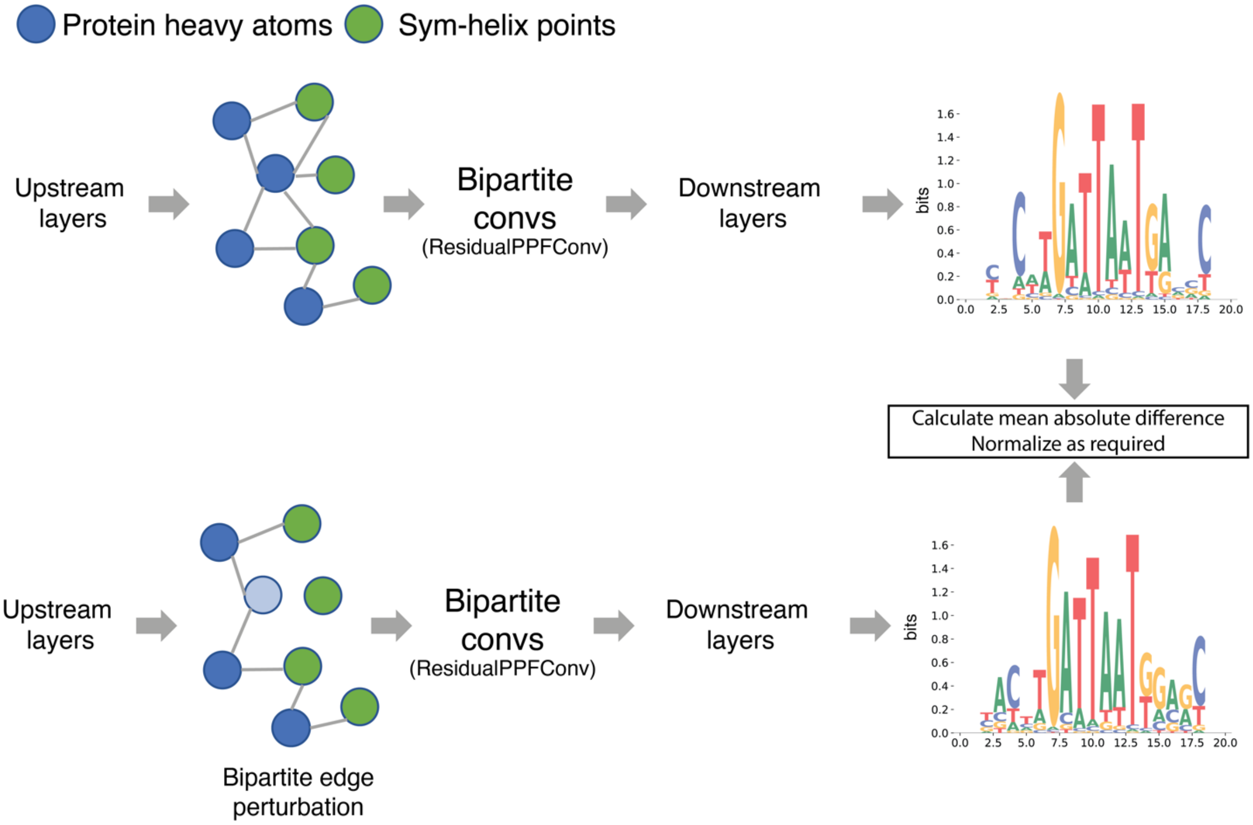

Fig. S4.

Schematic representation of bipartite edge-perturbation process. Blue circles denote protein heavy atoms. Green circles represent sym-helix points. In one forward pass, the output is calculated with all edges present. In another pass, edges corresponding to one protein heavy atom are excluded from the message-passing scheme, resulting in an alternate output. The difference between the two outputs can be quantified using the mean absolute difference measure and normalized as needed for interpretation purposes.

7. Additional discussion on behavior of metrics

To get a better perspective of the behavior of MAE metric, we demonstrated of how the MAE metric behaves for various different target PWM columns and possible predictions (Fig. S10a**)**. The predictions are of three forms, based on interpolated values of a variable $x \in [0,1]$. They are as follows: $[1-x, 0, x, 0]$, $\left[x, \frac{1-x}{3}, \frac{1-x}{3}, \frac{1-x}{3}\right]$, $[\frac{1-x}{3}, \frac{1-x}{3}, \frac{1-x}{3}, x]$. These demonstrations, although they do not form an exhaustive set, but give an idea of the behavior of the MAE metric. Based on these plots and taking into account the inherent performance limit computed for this metric, we can consider values less than 0.8 to be of reasonable agreement and below 0.6 to be in good agreement. Although, we do note that these values should not be set in stone as the problem in question is a regression problem as opposed to binary classification.

Some further thoughts on this matter: In theory, the uniform prediction [0.25, 0.25, 0.25, 0.25] can be regarded as a bad prediction, because a naive model can always predict such a value. This prediction will perform well for highly non-specific binders (e.g., cases like Fig. S8) but will fail for highly specific binders. However, the one-hot prediction [1,0,0,0] can also be a naïve (bad) prediction for this problem setting. It is the case when the sequence present in the co-crystal structure itself is the output, e.g., for the sequence ACG: [[1,0,0,0],[0,1,0,0],[0,0,1,0]]. For experimentally determined structures, this prediction will generally perform well, especially for highly specific binders, but will fail for nonspecific binders. Ultimately, we wanted to create a general model that can handle specific binders and non-specific binders. So, we need a target metric to strike a balance between both scenarios. Therefore, in our opinion, looking at the predictive performances in context of the alignment scores of the co-crystal structure derived sequence with the target PWM gives a clearer picture. This has now been added (for the benchmark set) to the manuscript as Fig. S10b.

We choose a continuous distance metrics like MAE over statistical measures like PCC (Pearson R) or SCC (Spearman R) because they are not very robust with only four (linearly dependent) points (well-known result in the statistics community[17,18]). In our observation, SCC can only take a few discrete values in this scenario and can sharply change for a very small change of values that change the rank order. The PCC metric, although it takes continuous values, is affected by similar non-intuitive situations. This is easy to demonstrate by considering the following three slightly altered predictions:

PCC([0.5,0.5,0,0], [**0.25,0.25,0.25,0.25**]) = Undefined
PCC([0.5,0.5,0,0], [**0.23,0.24,0.26,0.27]**) = -0.9487
PCC([0.5,0.5,0,0], [**0.27,0.26,0.24,0.23**]) = 0.9487
(calculated using *scipy.stats.pearsonr()* function)

Intuitively, all three predictions are of similar caliber. However, the PCC metric paints a dramatically different picture. MAE on the other hand produces values of 1, 1.06 and 0.94. This is much more nuanced and intuitive. We can also easily construct other non-intuitive situations for PCC. For example, if the target is [0.1,0.2,0.3,0.4], any monotonically increasing prediction will have a high PCC value.

PCC([0.1,0.2,0.3,0.4], **[0.001,0.002,0.003,0.994]**) = 0.776

This gives the impression that this prediction is quite good, while in reality, it is almost just a one-hot prediction. MAE on the other hand produces a value of 1.187 which depicts a bad prediction.

8. Additional details associated with application of DeepPBS on predicted structures

Running rCLAMPS: We ran the rCLAMPS model with default parameters provided by the model's authors according to instructions provided through their GitHub.

Running RoseTTAfoldNA (RFNA): We ran the RFNA model with default options and model weights (version: April 13, 2023 v0.2) as provided by the authors through their GitHub.

Trimming unstructured regions from full-length homeodomain (HD) sequences: For the analysis in Fig. 3e-i, full-length HD sequences were first trimmed to remove unstructured regions, while retaining the main Homeobox domain of interest (rCLAMPS also applies the same process in its pre-processing). This step was achieved by HMMERv3.4[19] using the 'homeobox.hmm' file provided by the rCLAMPS repository.

MM-PBSA energy calculation: For each PDB file, we generated a topology file and a run-parameter file using Gromacs 2020.3 to define the force fields amber14sb for protein and parmbsc1 for DNA. These files were used as input for g_mmpbsa to calculate the potential energy in a vacuum. The dielectric constant of the solute was set to 8.

Dataset: We obtained UniProt protein sequences for three different families, bZIP (homodimers), bHLH (homodimers), and homeodomain (HD) (heterodimers excluded), for cases with a corresponding unique JASPAR entry and no experimental structure for the complex. RFNA predictions that could be successfully processed by DeepPBS pre-processing steps were fed into DeepPBS for specificity prediction (n=49 for bHLH, n=50 for bZIP, n=236 for HD family members).

Choice of initial guess (IG) DNA: The IG DNA for the bHLH family was chosen as 'GCGCACCACGTGGTGCGC', which has a central E-box motif ('CACGTG') that is known[20] to be a bHLH family target. The IG DNA for the bZIP family was chosen as 'GCGCTGATGTCAGCGC' (based on human CREB1 motif MA0018.4). The IG DNA for the HD family was chosen as 'GCGTGTAAATGAATTACATGT', based on DNA from PDB ID 1APL.

Details on metric calculation for Fig. 3d: We calculated MAE (best full overlap) for predictions in Fig. 3d against corresponding JASPAR annotations. As a baseline (apart from random predictions drawn from uniform), we calculated the MAE (best full overlap) for the one-hot PWM determined by the IG DNA against the corresponding JASPAR annotation. For the bHLH and HD families, the IG DNA was closer to experimental data than to random baseline (Fig. 3d).

pLDDT score: (RFNA-predicted LDDT (Local Distance Difference Test)[21] score). The LDDT score measures the similarity between a predicted and reference structure. When predicting a complex structure, RFNA predicts an LDDT score (pLDDT). These pLDDT scores were shown[22] to be well correlated with the true LDDT of RFNA predictions. Thus, the pLDDT can be taken as a measure of quality of a complex generated by RFNA.

Comparison of DeepPBS and rCLAMPS: There are several qualitative advantages to the DeepPBS approach. First, rCLAMPS uses structural mapping for HD-DNA binding to predict specificity for a given HD sequence. This structural mapping leads to a specificity output of exactly six 6 base pairs (bp). DeepPBS is functionally not limited to only predicting a 6-base pair core and can predict preferences in the flanks (Fig. 3e). Second, rCLAMPS is restricted to predicting monomer preferences (although HD proteins can often bind as dimers; see, e.g., Fig. S6a). In contrast, DeepPBS is able to handle biological assemblies. Third, DeepPBS is not limited to a specific family. For quantitative comparison, we compare the aspects that are achievable by rCLAMPS, namely, 6-mer specificity predictions for monomer HD proteins.

Metric computation for comparison with rCLAMPS: We ran rCLAMPS (GitHub commit version 32a94edb65e87c6d038823dc34c4bcf6e1071b7b) on the set of monomer HD proteins (with unavailable complex structure, i.e. not part of RFNA or DeepPBS training). We computed the MAE (best overlap) values for rCLAMPS predictions against the corresponding JASPAR entry of experimental data, and compared these values to the MAE values of the best 6-mer overlap for DeepPBS predictions. In this case, for each datapoint, one of round 4-7 prediction was chosen. This choice was based on maximizing corresponding protein-DNA contact count (5 Å cutoff) of input RFNA predicted structure.

Folding benchmark set datapoints with RFNA: We refolded all the benchmark set datapoints with RFNA, for results presented in Fig. 3g. 108/130 RFNA predictions produced pre-processable results. Out of those, a few were not able to place the protein near the DNA helix properly. So, we filtered an atom-to-

atom contact count (within 5 Å) of greater than 500 contacts between protein and DNA to filter this set producing a set of size 98. The high confidence set among these are taken as predictions for which the RFNA pLDDT was greater that 0.9.

9. Molecular Dynamics (MD) simulation of Exd-Scr–DNA system

We conducted MD simulations on the *Drosophila* Extradenticle (Exd)-Sex combs reduced (Scr) system, with the dimer bound to its target DNA, using the co-crystal structure (PDB ID: 2R5Z). AlphaFold2 predictions of the proteins were aligned to the PDB structure to create an initial structure of the simulation. This process aided in filling in the missing linker residues in the biological assembly. The simulation was executed using the Gromacs[23] 2020.3 software package. Protein interactions were modeled with the amber14sb[24] force field, and DNA interactions were modeled with the parmbsc1[25] force field. The pdb2gmx program from Gromacs was used to generate topological information for the simulation. The -his flag was used to protonate both Nδ and Nϵ atoms of the His-12 residue for the system with protonated His-12. All complexes were solvated using the explicit TIP3P water model. The negative net charge of the Exd-Hox-DNA complex was neutralized by adding positively charged Na+ counterions, along with negatively charged Cl- counterions, to reach a final NaCl concentration of 150 mM that approximates the physiological salt concentration. The GROMACS 2020.3 genion program was used to place these counterions throughout the box.

The protein-DNA complex was energy-minimized with steep descent energy minimization for 2,000 steps to relax the structure and remove any steric clashes. Next, we performed three rounds of gradual NVT (constant Number of particles, Volume, and Temperature) equilibration for 10 ps to slowly heat the prepared system to 300 K and 1 round of NPT (constant Number of particles, Pressure, and Temperature) to equilibrate the pressure of the system to 1 bar for 700 ps. These equilibration rounds were used to adjust the whole system to biological conditions before starting the production simulation. The production simulation for the system was run for 300 ns in the isobaric–isothermal ensemble, where the pressure was maintained at 1 bar and temperature at 300 K. The integration time step of 2 fs was used for all calculations. The Verlet cutoff scheme was used for all calculations. Long-range electrostatic interactions were computed using the Particle Mesh Ewald method[26] from the GROMACS v2020.3 package, with a 12 Å cutoff. Nonbonded van der Waals interactions were calculated with a 12 Å cutoff. The LINCS[27] algorithm from the GROMACS v2020.3 package, was employed to constrain all bonds. The MD simulation trajectories were generated as part of a recent study exploring effects of minor groove linker histidine protonation[28].

10. Additional details on application of DeepPBS to MD simulation of Exd-Scr–DNA system

Owing to a fast inference time, DeepPBS can be used to analyze molecular simulation trajectories. We demonstrated how the protein heavy atom-level interpretability allows automatic detection of conformational changes in the protein-DNA interface. We applied DeepPBS to an MD simulation of the well-studied Exd-Scr-DNA system (Fig. S7a) (PDB ID: 2R5Z)[29–32]. Details of the simulation method are provided in Supplementary Section 9. By computing the DeepPBS prediction over the trajectory, (Supplementary Video S1) consistent with the known binding specificity[29] of the system. The simulation trajectory was divided into 3,000 snapshots (0.1 ns apart), and the DeepPBS ensemble was applied to predict binding specificity for each snapshot. Relative importance (RI) scores were calculated for each heavy atom within 5 Å of DNA, followed by computation of max-aggregated residue RI scores. Fig. S6a shows the initial structure of the simulation, with the locations of some residues of interest marked. Residues Arg5 and His-12 of the Scr protein contribute to minor groove narrowing through electrostatic interactions, which play a crucial role in determining binding specificity[33].
Residues Arg58, Ile57, and Lys61 on the Exd protein interact with the major groove, driving specificity through hydrogen bonding and van der Waals interactions. In the simulation, residues Arg2, Arg3, and Arg5 on Exd contact with the flanking sequences.

Variation of RI of the residues discussed earlier are shown in Fig. S6b,d,f. Supplementary Video 1 shows a concurrent view of changes in the network prediction as the simulation progressed, along with

corresponding changes in the heavy atom RI score. Throughout the trajectory, Arg5 and His-12 on Scr consistently interact in the minor groove to drive protein-DNA binding specificity (Fig. S6e). Our model assigns stable RI scores to these residues (Fig. S6d). Arg58 strongly drives specificity by contacting G in the major groove, forming a bidentate hydrogen bond. However, after ~100 ns of simulation, the Exd recognition helix moves closer to the DNA major groove, leading to rotation of Arg58 (Fig. S6f, g) and causing a loss of strong specificity for G. Lys61 intermittently contacts the DNA through strong electrostatic interactions, leading to a gain in RI (Fig. S6f, g).

RI scores assigned by our end-to-end deep-learning model offer an efficient alternative to traditional energy calculations, which require meticulous force-field design and energy computations. In the case of residues Arg2, Arg5, and Arg3 at the terminal loop region of the Exd protein, temporal changes in RI scores (Fig. S6b) strongly correspond to conformational changes of these residues over the simulation trajectory, as highlighted in Fig. S6c. Arg2 forms a bidentate H-bond with G (~40 ns to 100 ns), which appears in DeepPBS predictions as highly specific for C (Fig. S6b). Arg5 interacts with an adjacent minor groove for most of the trajectory; however, it deviates away from the minor groove after ~210 ns, and a corresponding reduction in RI is observed. This demonstrates the ability of our deep-learning model to capture the dynamic behavior of residues and their interactions with the DNA.

DeepPBS has demonstrated its robustness and adaptability in response to both small dynamical fluctuations and conformational changes. Although the model was trained on snapshot structures and experimental PWMs, its predictions and RI scores are well-regularized and versatile, making it suitable for automated analysis of MD trajectories and designed protein-DNA complexes. These factors make DeepPBS a valuable tool for researchers working in the field of protein-DNA interactions, enabling deeper understanding and insights into the behavior of these complex molecular systems.

11. Measures ensuring prevention of overfitting to protein sequences

We took several steps to prevent the model to be overfit on protein sequences. The cross-validation fold creation script (see Code Availability) took care to keep members of the same cluster in the same fold (except a handful (<4%) got split randomly to keep the fold sizes same). Full description of these splits is available (see Data Availability) and the code for this process is available (see Code Availability). In addition, no more than five samples were chosen per cluster into a fold. This ensures prevention of over representation. Furthermore, the input to DeepPBS is purely structural and physico-chemical, and does not contain the sequence representation. These structures may demonstrate different spatial conformations interacting with potentially different DNA sequence/shape and randomly picked target PWM from JASPAR or HOCOMOCO. These guardrails in architecture and all of these variations, even for similar protein sequences, makes the model less prone to overfit on protein sequences.

12. Details on outliers seen on the benchmark set performance presented in Fig. 2a

The outlier for the DeepPBS (Groove Readout) model is a TATA-box binding protein (TBP) bound to nucleosome bound-DNA (PDB ID: 7OH9). It is understandable that the ‘groove readout’ model will fail for this structure simply because TBP-DNA binding is known to be a primarily ‘shape readout’ driven process depending on the strong bendability and high conformational flexibility of the TATA motif[34]. Other than data quality limitations, another form of data limitation can be representation. For example, carboxylic acid side chains (glutamic and aspartic acids) are generally rare in biological DNA binding domains and hence in DeepPBS training data. A synthetic protein chemist should be weary of this fact, while designing domains with these residues.

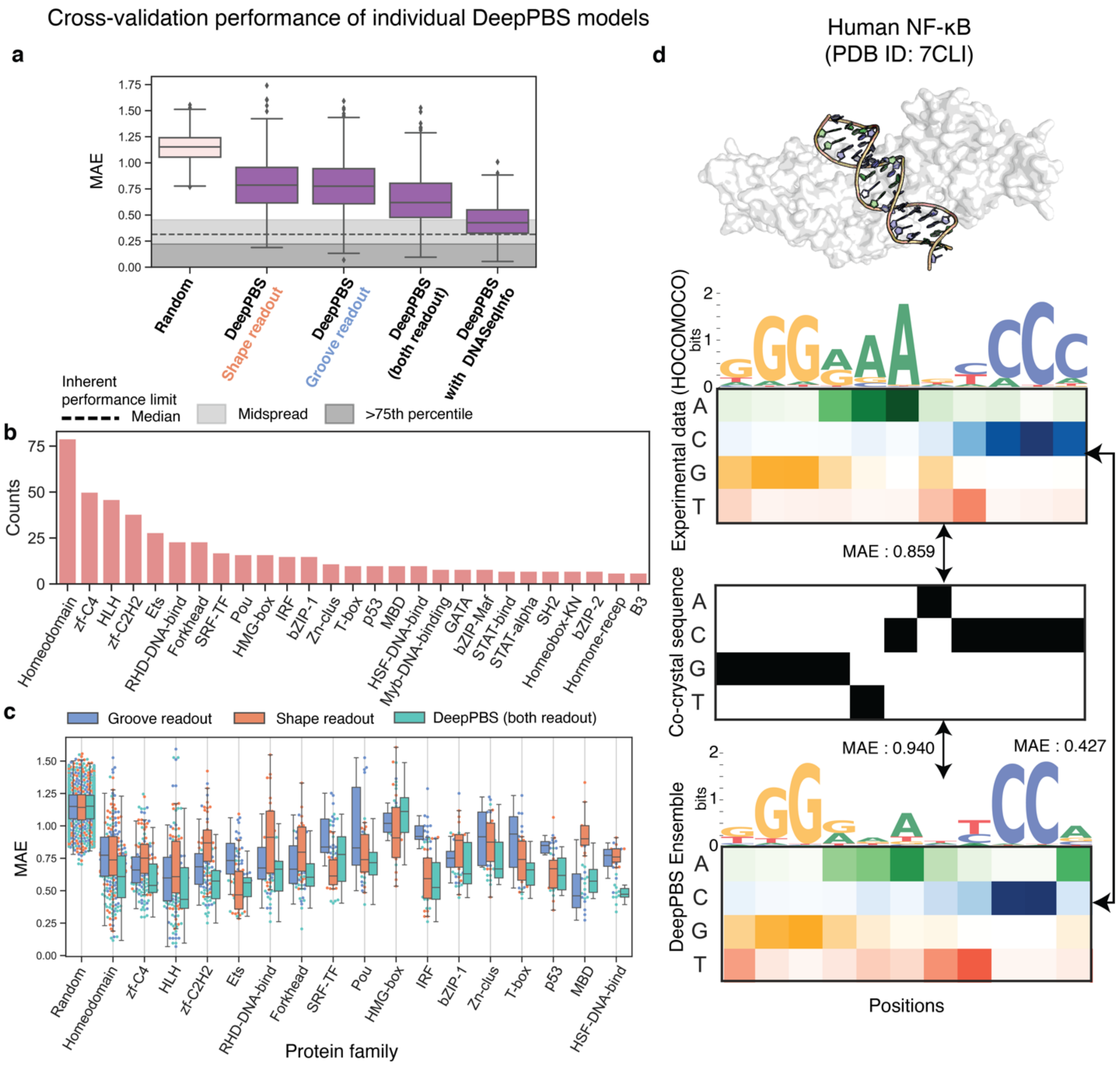

Fig. S5.

Cross-validation performance of DeepPBS for predicting binding specificity across protein families on experimentally determined structures. (a) Cross-validation performance of individual trained models for the DeepPBS model and its variations (biological assemblies corresponding to *n*=523 protein chains (for each box plot)) **(b)** Abundance of various protein families (PFAM annotations) in cross-validation dataset (counts>8). **(c)** Cross-validation performance of DeepPBS, along with 'groove readout' and 'shape readout' variations, across protein families (counts > 8). (Biological assemblies corresponding to *n* protein chains (for each family), where *n* is as described in **(b),** total unique *n*=523) **(d)** Example DeepPBS ensemble prediction on NF-κB biological assembly (sampled in benchmark set) containing non-optimal DNA sequence. DeepPBS ensemble prediction on NF-κB biological assembly for human (NFKB2, UniProt ID Q00653) is shown in the benchmark dataset. The colored heatmaps (top and bottom) encode probabilities of base identity. The back and white heatmap (center) represents the co-crystal structure. Although the co-crystal structure derived DNA sequence is not of the highest affinity (judging by experimental data from HOCOMOCO), our prediction can circumvent this issue and predict binding specificity levels that are much closer to those observed experimentally. For box plots in **(a)** and **(c)**, lower limit represents lower quartile, middle line represents median, and upper limit represents upper quartile. Whiskers do not include outliers.

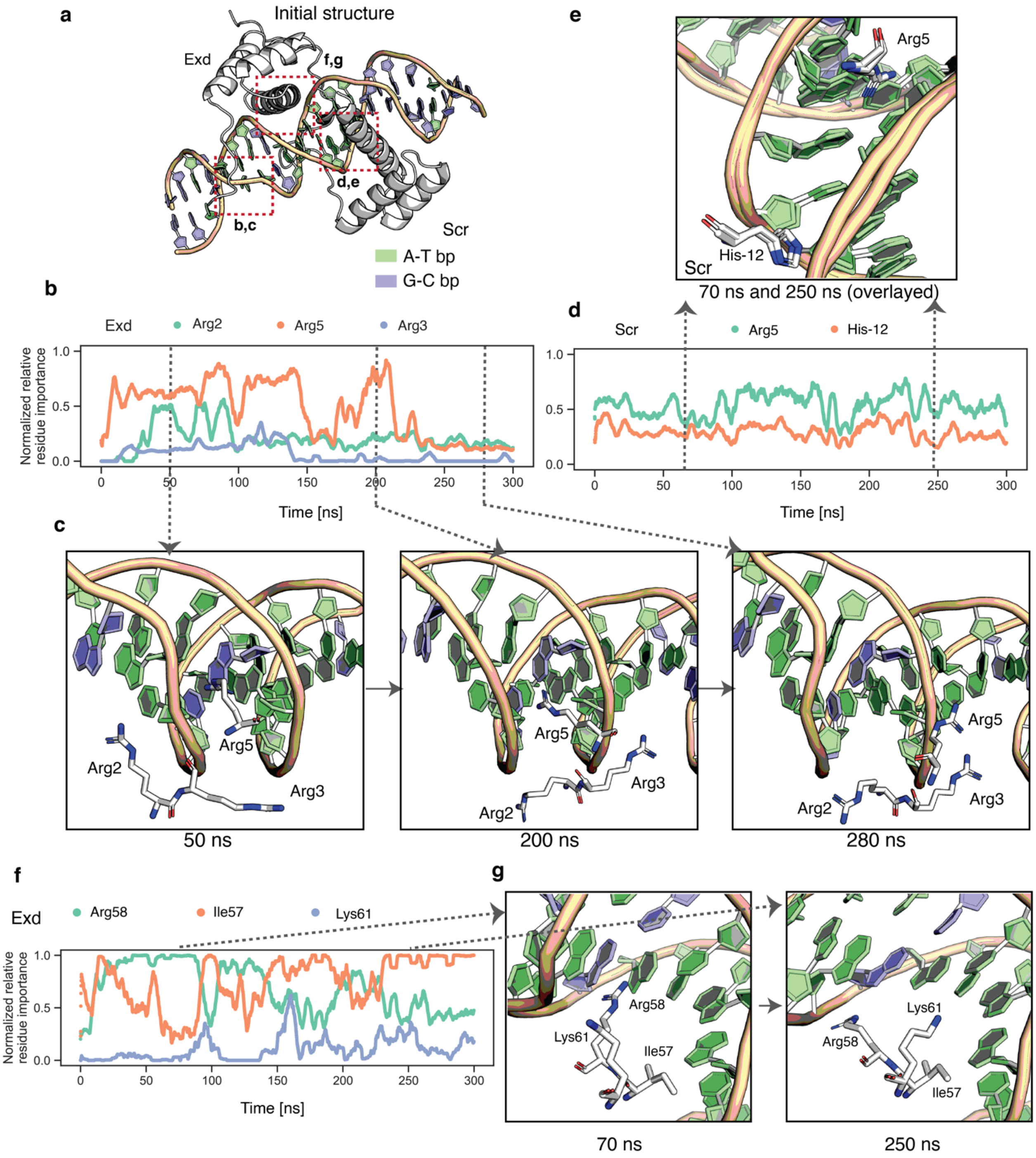

Fig. S6.

Application of DeepPBS to MD simulation of AlphaFold2- and PDB (2R5Z)-based modeled complex of Exd-Scr system. (a) Initial structure of simulation. Locations of residues of interest are marked. **(b)** Residue-level RI score over time (averaged per 5 ns window) for residues involved in Exd-DNA flank ineraction. **(c)** Snapshot of interactions by Exd Arg2, Arg3, and Arg5 at 50 ns, 200 ns, and 280 ns, respectively. **(d)** Residue-level RI score over time (averaged 5 ns window) for residues involved in Scr-DNA minor groove interaction. **(e)** Snapshot of interactions by Scr Arg5 and His-12 at 70 ns and 250 ns, respectively. **(f)** Residue-level RI score over time (averaged per 5 ns window) for residues involved in Scr-DNA major groove interaction. **(g)** Snapshot of interactions by Exd Arg58, Lys6, and Ile57 at 70 ns and 250 ns.

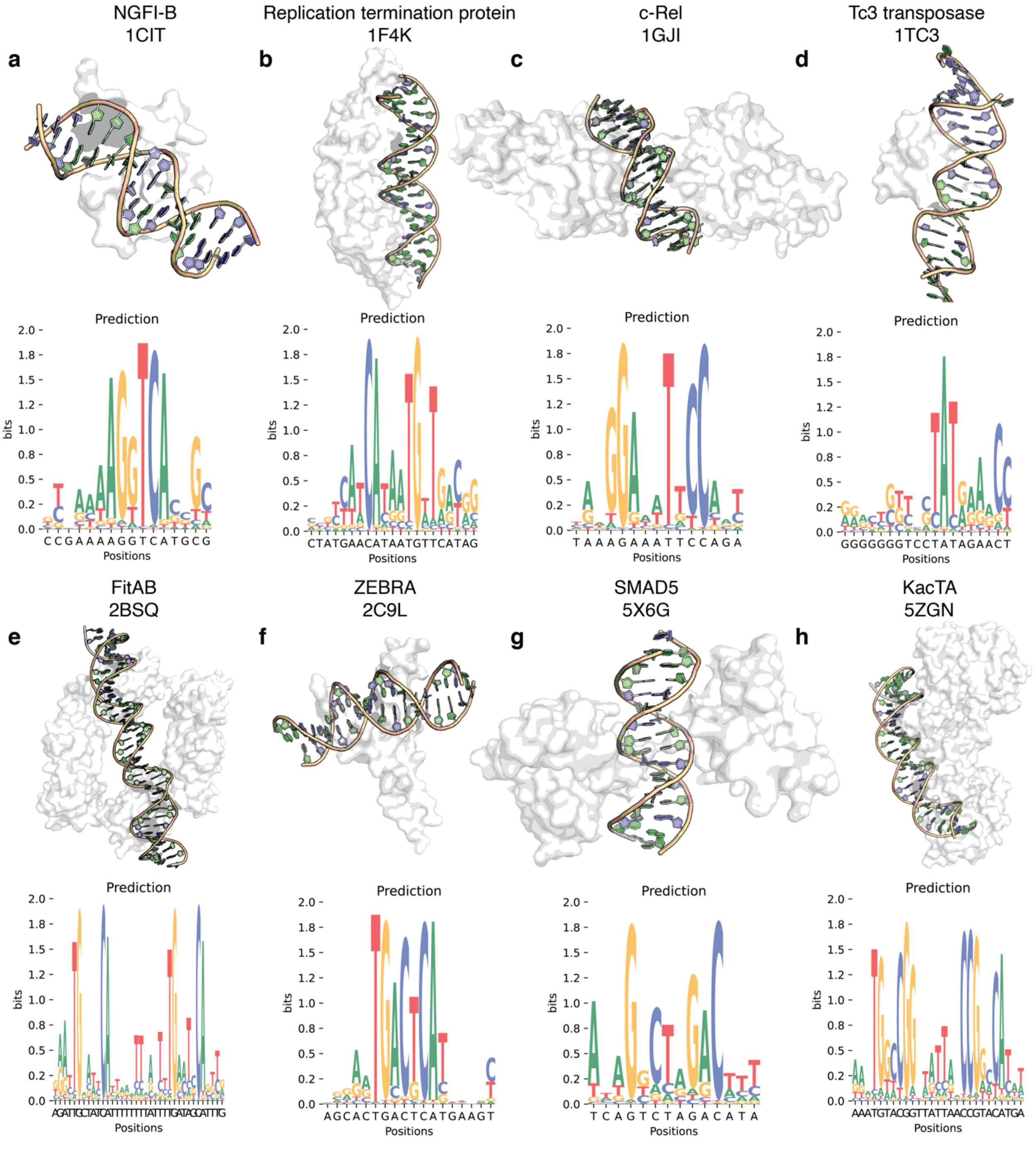

Fig. S7.

Example DeepPBS ensemble predictions on structures of specific DNA binders. Specificity data were unavailable on JASPAR/HOCOMOCO. **(a)** Monomeric orphan nuclear receptor NGFI-B, **(b)** replication termination protein in bacteria, **(c)** proto-oncogene product c-Rel, **(d)** Tc3 transposase bound to transposon DNA, **(e)** Fitab protein from *Neisseria Gonorrhoeae*, **(f)** Epstein-Barr virus ZEBRA protein, **(g)** Smad5-MH1 protein/ palindromic SBE DNA complex, and **(h)** DUF1778 domain-containing kacTA protein.

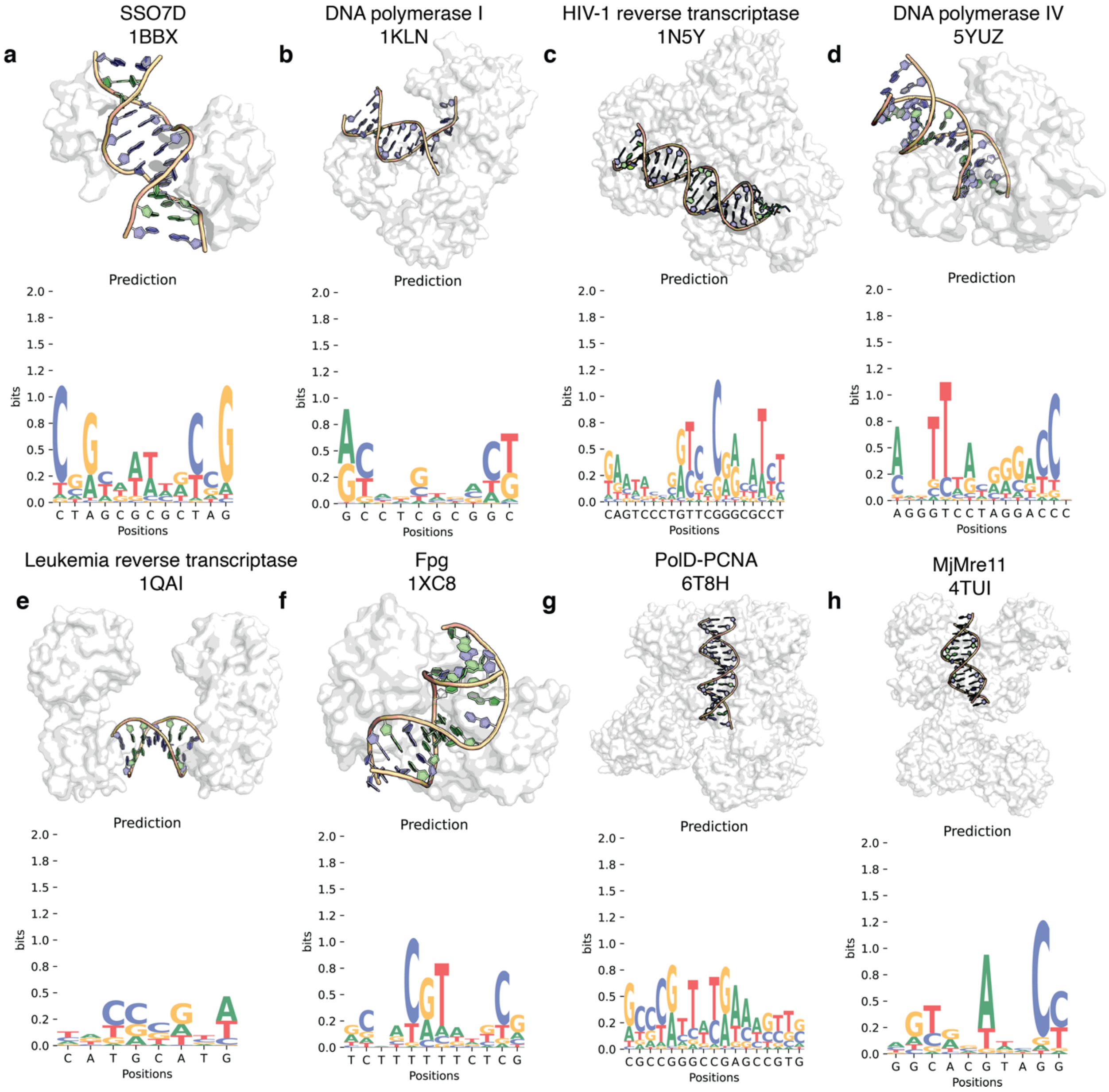

Fig. S8.

Example DeepPBS ensemble predictions on structures of non-specific DNA binders. (a) Sso7D-DNA complex, **(b)** DNA polymerase I Klenow fragment, **(c)** HIV-1 reverse transcriptase with pre-translocation and post-translocation AZTMP-terminated DNA, **(d)** DNA polymerase IV, **(e)** Moloney murine leukemia virus reverse transcriptase, **(f)** DNA repair enzyme formamidopyrimidine-DNA glycosylase (Fpg), **(g)** DNA polymerases in complex with proliferative cell nuclear antigen (PCNA), and **(h)** DNA damage sensing enzyme *Methanococcus jannaschii* Mre11 (MjMre11).

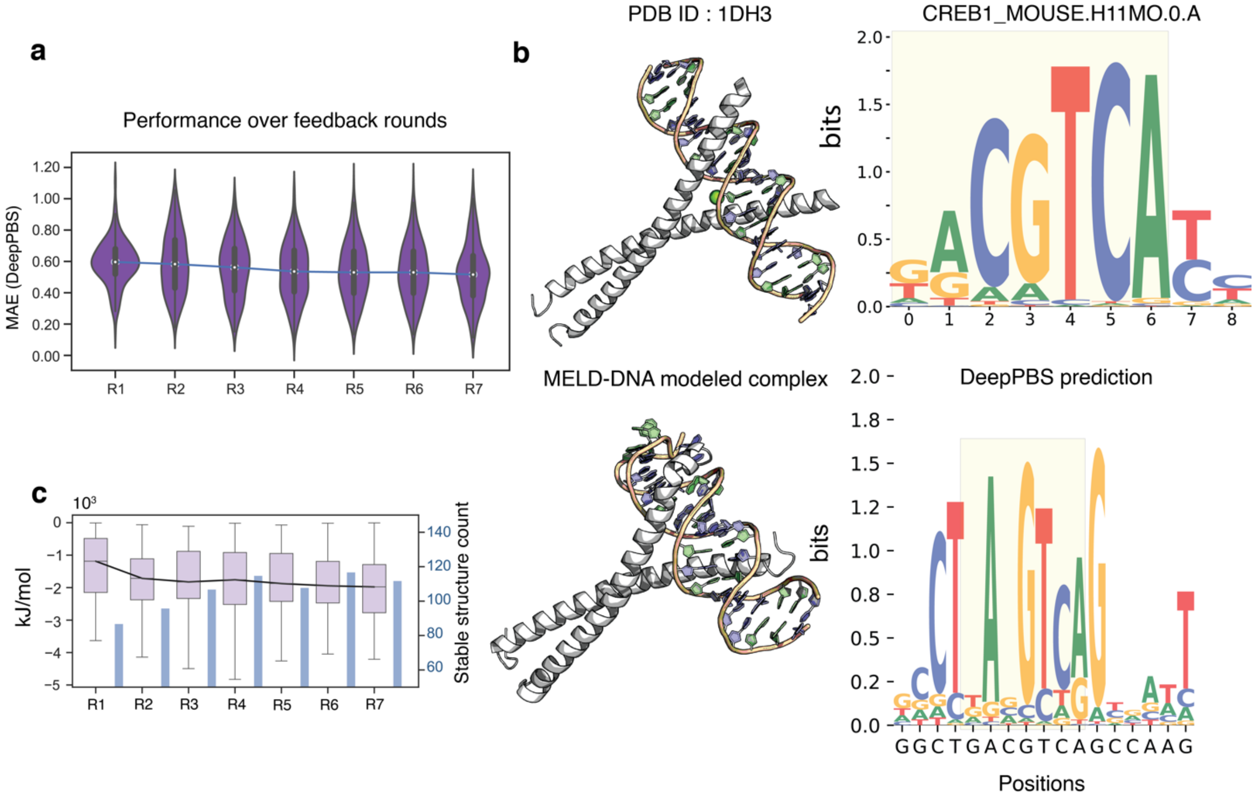

Fig. S9.

Application of DeepPBS on modeled structures. (a) Performance of DeepPBS (best 6-mer overlap) improves as the feedback loop progresses (corresponding to Fig. 3f) in conjunction with improvement in RFNA complex design. (*n* = 236 predicted assemblies) **(b)** Example application of DeepPBS on mouse CREB1 dimer bound to DNA modeled by MELD-DNA (as provided by their authors). **(c)** Calculated MM-PBSA (Supplementary Section 7) vacuum energy distribution for stable RFNA predictions (<0 kJ/mol) and corresponding counts over rounds 1–7 (for *n* predicted assemblies for each boxplot, where *n* is same as stable structure count (corresponding bar plot)). For box plots in **(a)** and **(c)**, lower limit represents lower quartile, middle point/line represents median, and upper limit represents upper quartile.

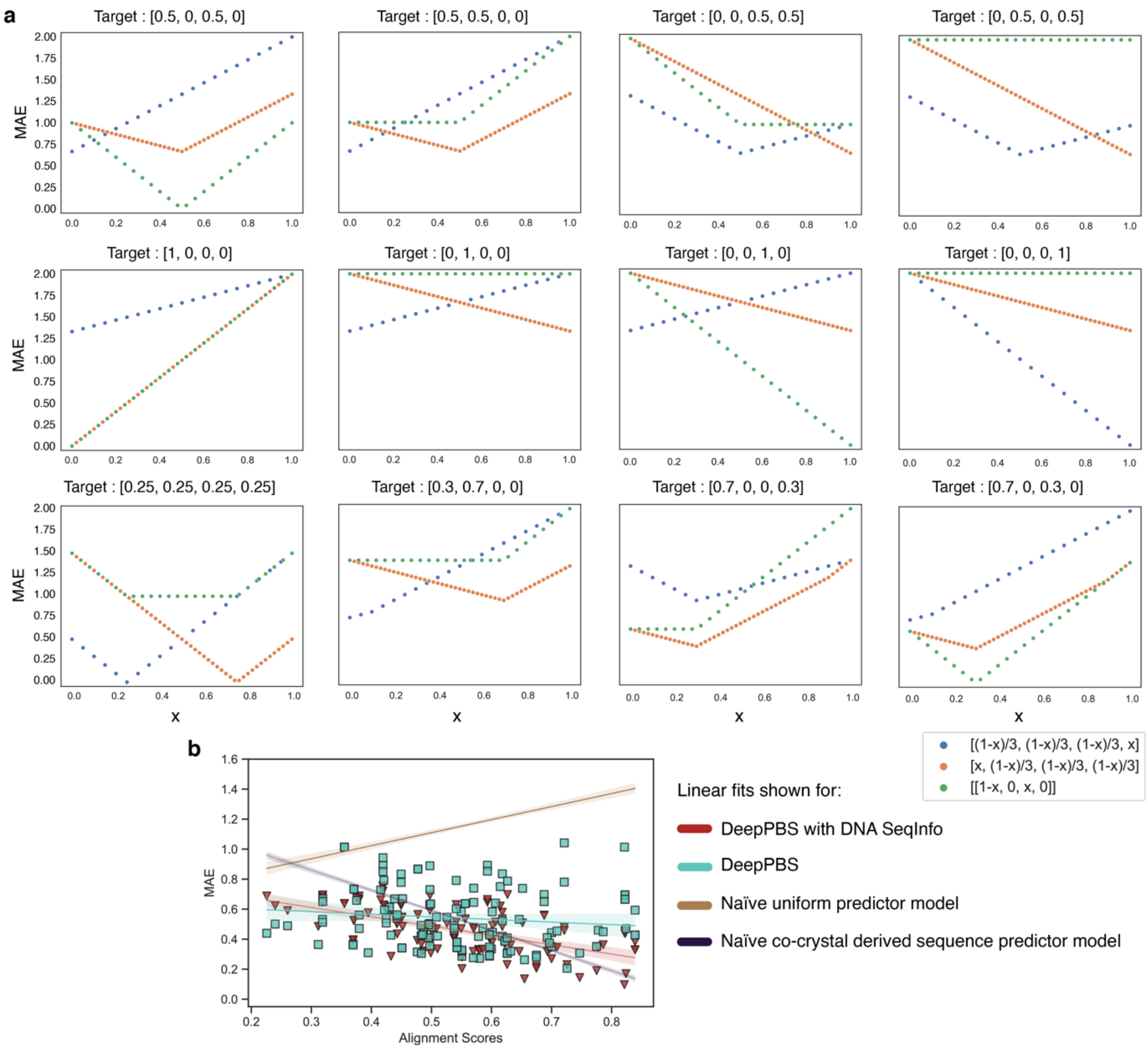

Fig. S10.

Behavior of metric for different target PWM and interpolated predictions. (a) Demonstrations of how the MAE metric behaves for various different target PWM columns and possible predictions (Fig. S10a). The predictions are of three forms, based on interpolated values of a variable $x \in [0,1]$ as $[1-x, 0, x, 0]$, $\left[x, \frac{1-x}{3}, \frac{1-x}{3}, \frac{1-x}{3}\right]$, $[\frac{1-x}{3}, \frac{1-x}{3}, \frac{1-x}{3}, x]$. Although not an exhaustive set, we hope it helps the reader put into context the behavior of this metric. **(b)** DeepPBS and DeepPBS with DNA SeqInfo performance on benchmark set in context of two naïve models (uniform predictor and co-crystal structure derived sequence predictor). Lines indicates linear regression fit. Light shaded regions indicate corresponding 95% confidence interval computed via bootstrapping mean.

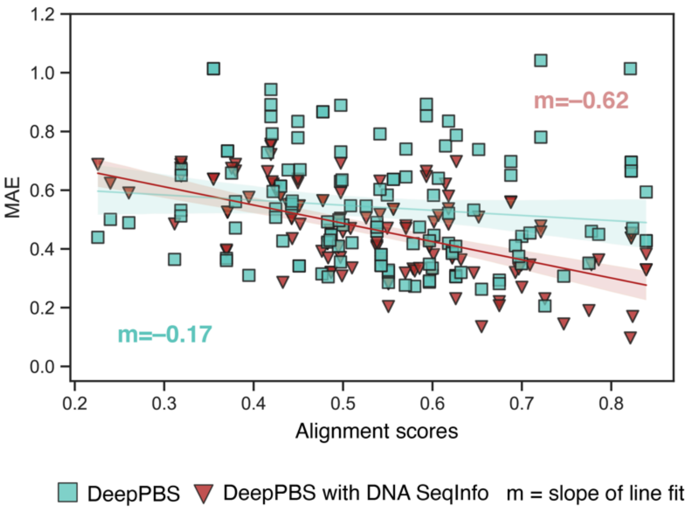

Fig. S11.

MAE equivalent of Fig. 2b showing the bias of the DeepPBS with DNA SeqInfo model compared to the DeepPBS model towards the alignment score of co-crystal DNA structure derived sequence and target PWM. Lines indicates linear regression fit. Light shaded regions indicate corresponding 95% confidence interval computed via bootstrapping mean.

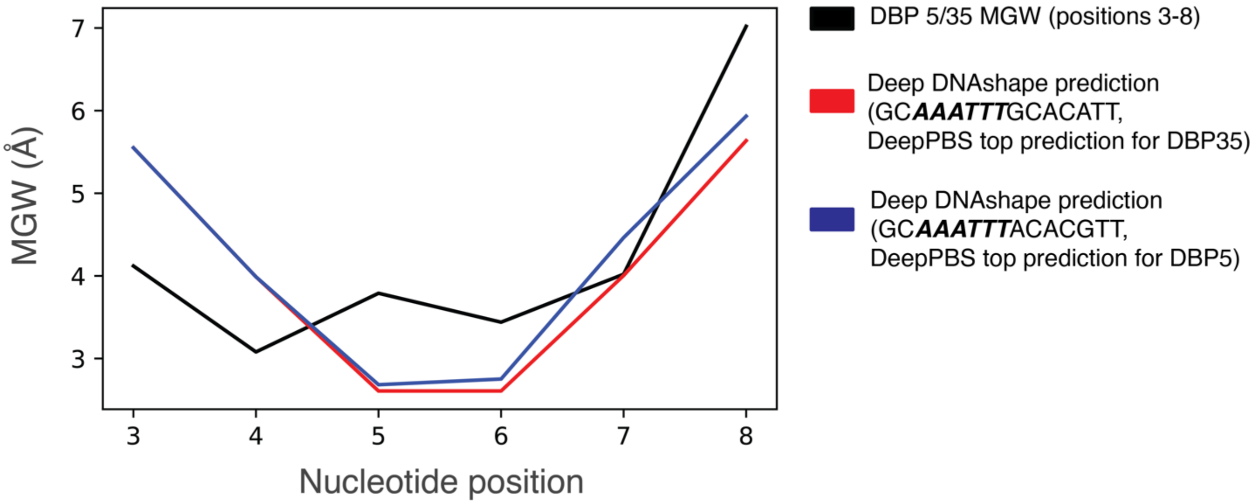

Fig. S12.

Deep DNAshape[36] prediction of the minor groove width (MGW) profile for the sequence AAATTG (top DeepPBS prediction for the designed model DBP35 and DBP5, positions 3–8) compared to the actual MGW of the DNA in these designed complexes.

DeepPBS	$\Delta\Delta G$ (kcal/mol)	PDB ID	Residue
3.4246	2.62	1b3t	Y518
0.0973	0.17	1fos	K148
0.1073	0.17	1fos	K153
3.0678	1.04	1fos	R155
0.1036	0.34	1fos	R158
0.0782	0.04	1fos	R272
2.8199	0.84	1hcq	E25
0.7292	0.92	1hcq	H18
4.2104	1.21	1hcq	K28
2.8322	1.24	1hcq	K32
1.0187	1.22	1hcq	Y19
0.6486	0.7	1j5n	K22
0.6486	0.7	1j5n	K22
0.5429	0.5	1j5n	K53
1.0272	0.4	1j5n	K60
0.9116	0.4	1j5n	K78
1.0853	0.2	1j5n	K85
3.2762	0.5	1j5n	M29
0.2997	0.7	1j5n	N33
0.9606	0.8	1j5n	R36
0.7074	0.7	1j5n	R40
3.751	0.9	1j5n	Y28
0.7891	0.2	1j5n	Y81
1.7017	0.2	1mse	S187
1.1549	0.7	1tn9	K21
3.2913	1.36	1tn9	K28
0.2911	1.33	1tn9	K54
2.8115	0.43	1tn9	R20
0.996	1.22	1tn9	R24
2.6845	1.17	1tn9	R55
0.0579	0.75	1tn9	R5
0.5691	0.04	1tn9	T15
0.3096	0.44	1tn9	W42
2.596	1.51	1tn9	Y40
0.5089	0.2725	2mxf	K102
0.6247	0.6225	2mxf	K105
1.1177	0.7518	2mxf	K108
0.5467	0.283	2mxf	K81
2.0751	0.2967	2mxf	K97
4.1414	0.7725	2mxf	N100
4.454	1.3382	2mxf	R80
3.5985	2.0467	3ufd	R46
0.6796	0.971	3ufd	S52
2.6846	1.4177	3ufd	Y37
3.7829	2.2518	4bnc	R391

Table S1.
Data presented in Fig. 4h along with corresponding PDB IDs and target residues.

Supplementary References

1. Berman, H. M. *et al.* The protein data bank. *Nucleic Acids Res.* **28**, 235–242 (2000).
2. Castro-Mondragon, J. A. *et al.* JASPAR 2022: the 9th release of the open-access database of transcription factor binding profiles. *Nucleic Acids Res.* **50**, D165–D173 (2022).
3. Kulakovskiy, I. V *et al.* HOCOMOCO: towards a complete collection of transcription factor binding models for human and mouse via large-scale ChIP-Seq analysis. *Nucleic Acids Res.* **46**, D252–D259 (2018).
4. Park, P. J. ChIP-seq: Advantages and challenges of a maturing technology. *Nat. Rev. Genet.* **10**, 669–680 (2009).
5. Lu, X.-J., Bussemaker, H. J. & Olson, W. K. DSSR: An integrated software tool for dissecting the spatial structure of RNA. *Nucleic Acids Res.* **43**, e142–e142 (2015).
6. Fu, L., Niu, B., Zhu, Z., Wu, S. & Li, W. CD-HIT: Accelerated for clustering the next-generation sequencing data. *Bioinformatics* **28**, 3150–3152 (2012).
7. Peng, Y., Sun, L., Jia, Z., Li, L. & Alexov, E. Predicting protein–DNA binding free energy change upon missense mutations using modified MM/PBSA approach: SAMPDI webserver. *Bioinformatics* **34**, 779–786 (2018).
8. Chiu, T. P., Rao, S. & Rohs, R. Physicochemical models of protein–DNA binding with standard and modified base pairs. *Proc. Natl. Acad. Sci. USA* **120**, (2023).
9. Lavery, R. & Sklenar, H. The definition of generalized helicoidal parameters and of axis curvature for irregular nucleic acids. *J. Biomol. Struct. Dyn.* **6**, 63–91 (1988).
10. Lu, X.-J. & Olson, W. K. 3DNA: a versatile, integrated software system for the analysis, rebuilding and visualization of three-dimensional nucleic-acid structures. *Nat. Protoc.* **3**, 1213–1227 (2008).
11. Atchley, W. R., Zhao, J., Fernandes, A. D. & Drüke, T. Solving the protein sequence metric problem. *Proc. Natl. Acad. Sci. USA* **102**, 6395–6400 (2005).
12. Xie, T. & Grossman, J. C. Crystal graph convolutional neural networks for an accurate and interpretable prediction of material properties. *Phys. Rev. Lett.* **120**, 145301 (2018).
13. Agarap, A. F. Deep learning using rectified linear units (relu). *arXiv preprint arXiv:1803.08375* (2018).
14. Deng, H., Birdal, T. & Ilic, S. PPFNet: Global context aware local features for robust 3D point matching. *IEEE/CVF Conf. Comput. Vision Pattern Recogn.* 195–205 (2018).
15. Bridle, J. S. Probabilistic interpretation of feedforward classification network outputs, with relationships to statistical pattern recognition. in *Neurocomputing* 227–236 (Springer Berlin Heidelberg, 1990).
16. Kingma, D. P. & Ba, J. Adam: A Method for Stochastic Optimization. *ArXiv* **1412.6980**, (2017).
17. Bonett, D. G. & Wright, T. A. Sample size requirements for estimating pearson, kendall and spearman correlations. *Psychometrika* **65**, 23–28 (2000).
18. McIntyre, F. & David, F. N. Tables of the ordinates and probability integral of the distribution of the correlation coefficient in small samples. *J. Am. Stat. Assoc.* **33**, 751 (1938).
19. Finn, R. D., Clements, J. & Eddy, S. R. HMMER web server: interactive sequence similarity searching. *Nucleic Acids Res.* **39**, W29–W37 (2011).
20. de Martin, X., Sodaei, R. & Santpere, G. Mechanisms of binding specificity among bHLH transcription factors. *Int. J. Mol. Sci.* **22**, 9150 (2021).
21. Mariani, V., Biasini, M., Barbato, A. & Schwede, T. lDDT: a local superposition-free score for comparing protein structures and models using distance difference tests. *Bioinformatics* **29**, 2722–2728 (2013).
22. Baek, M., Mchugh, R., Anishchenko, I., Baker, D. & Dimaio, F. Accurate prediction of nucleic acid and protein-nucleic acid complexes using RoseTTAFoldNA. *Nat. Methods* **21**, 117–121 (2024).
23. Abraham, M. J. *et al.* GROMACS: High performance molecular simulations through multi-level parallelism from laptops to supercomputers. *SoftwareX* **1–2**, 19–25 (2015).
24. Maier, J. A. *et al.* ff14SB: Improving the accuracy of protein side chain and backbone parameters from ff99SB. *J. Chem. Theory Comput* .**11**, 3696–3713 (2015).
25. Ivani, I. *et al.* Parmbsc1: a refined force field for DNA simulations. *Nat Methods* **13**, 55–58 (2016).
26. Darden, T., York, D. & Pedersen, L. Particle mesh Ewald: An *N* ·log(*N*) method for Ewald sums in large systems. *J. Chem. Phys.* **98**, 10089–10092 (1993).
27. Hess, B., Bekker, H., Berendsen, H. J. C. & Fraaije, J. G. E. M. LINCS: A linear constraint solver for molecular simulations. *J. Comput. Chem.* **18**, 1463–1472 (1997).
28. Jiang, Y., Chiu, T. P., Mitra, R. & Rohs, R. Probing the role of the protonation state of a minor groove-linker histidine in Exd-Hox–DNA binding. *Biophys. J.* **123**, 248–259 (2024).
29. Abe, N. *et al.* Deconvolving the recognition of DNA shape from sequence. *Cell* **161**, 307–318 (2015).

30. Chiu, T. P., Li, J., Jiang, Y. & Rohs, R. It is in the flanks: Conformational flexibility of transcription factor binding sites. *Biophys. J.* **121**, 3765–3767 (2022).
31. Ghoshdastidar, D. & Bansal, M. Flexibility of flanking DNA is a key determinant of transcription factor affinity for the core motif. *Biophys. J.* **121**, 3987–4000 (2022).
32. Slattery, M. *et al.* Cofactor binding evokes latent differences in DNA binding specificity between Hox proteins. *Cell* **147**, 1270–1282 (2011).
33. Joshi, R. *et al.* Functional specificity of a Hox protein mediated by the recognition of minor groove structure. *Cell* **131**, 530–543 (2007).
34. Tolić-Nørrelykke, S. F., Rasmussen, M. B., Pavone, F. S., Berg-Sørensen, K. & Oddershede, L. B. Stepwise bending of DNA by a single TATA-box binding protein. *Biophys. J.* **90**, 3694–3703 (2006).
35. Lavery, R. & Sklenar, H. Defining the structure of irregular nucleic acids: Conventions and principles. *J. Biomol. Struct. Dyn.* **6**, 655–667 (1989).
36. Li, J., Chiu, T. P. & Rohs, R. Predicting DNA structure using a deep learning method. *Nat. Commun.* **15**, 1243 (2024).
