# Supplementary material for: Geometric deep learning of protein–DNA binding specificity
Source: Nat Methods. 2024 Aug 5;21(9):1674–83. doi: 10.1038/s41592-024-02372-w (PMC11399107; doi:10.1038/s41592-024-02372-w)
Supplement: Supplementary file 2 — Reporting Summary [file 41592_2024_2372_MOESM2_ESM.pdf]

nature portfolio

Corresponding author(s): Remo Rohs

Last updated by author(s): Apr 24, 2024

Reporting Summary

Nature Portfolio wishes to improve the reproducibility of the work that we publish. This form provides structure for consistency and transparency in reporting. For further information on Nature Portfolio policies, see our Editorial Policies and the Editorial Policy Checklist.

Statistics

For all statistical analyses, confirm that the following items are present in the figure legend, table legend, main text, or Methods section.

n/a	Confirmed	
☐	☒	The exact sample size (*n*) for each experimental group/condition, given as a discrete number and unit of measurement
☒	☐	A statement on whether measurements were taken from distinct samples or whether the same sample was measured repeatedly
☐	☒	The statistical test(s) used AND whether they are one- or two-sided<br>*Only common tests should be described solely by name; describe more complex techniques in the Methods section.*
☒	☐	A description of all covariates tested
☒	☐	A description of any assumptions or corrections, such as tests of normality and adjustment for multiple comparisons
☐	☒	A full description of the statistical parameters including central tendency (e.g. means) or other basic estimates (e.g. regression coefficient) AND variation (e.g. standard deviation) or associated estimates of uncertainty (e.g. confidence intervals)
☒	☐	For null hypothesis testing, the test statistic (e.g. *F*, *t*, *r*) with confidence intervals, effect sizes, degrees of freedom and *P* value noted<br>*Give P values as exact values whenever suitable.*
☒	☐	For Bayesian analysis, information on the choice of priors and Markov chain Monte Carlo settings
☒	☐	For hierarchical and complex designs, identification of the appropriate level for tests and full reporting of outcomes
☐	☒	Estimates of effect sizes (e.g. Cohen's *d*, Pearson's *r*), indicating how they were calculated

Our web collection on statistics for biologists contains articles on many of the points above.

Software and code

Policy information about availability of computer code

Data collection	Data was downloaded from publicly available datasets. (PDB, UniProtKB, JASPAR2022, HOCOMOCOv11, SAMPDI, MELD-DNA)
Data analysis	All core DeepPBS (https://github.com/timkartar/DeepPBS) code was written in python3.9+ with various pythonic dependencies (full list available at https://github.com/timkartar/DeepPBS/blob/main/deeppbs_linux.yml). Packages used for geometric deep learning are pytorch1.12+ and torch-geometric(pyg v2.0+). Data analysis and visualization was carried out in same environment. Structure visualizations presented in the manuscript are done using PyMOL2.5. 3DNAv2.3 and Curves5 was used in pre-processing steps (executables are provided https://github.com/timkartar/DeepPBS/tree/main/dependencies/bin). CD-HITv4.8.1 was used for protein sequence clustering. RFNA v0.2 was used for complex structure prediction. MD simulation was performed using Gromacs2020.3, LINCS algorithm included with Gromacs2020.3 was employed to constrain all bonds. HMMERv3.4 was used for homeobox detection. https://github.com/jlwetzel-slab/rCLAMPS and corresponding instructions were used for predictions by rCLAMPS (github commit version 32a94edb65e87c6d038823dc34c4bcf6e1071b7b) method. Installable source code, pre-trained models, associated guidelines and various custom scripts can be found at https://github.com/timkartar/DeepPBS. The implementation is also available via a Code Ocean capsule at https://doi.org/10.24433/CO.0545023.v2. In addition, DeepPBS is accessible as a web server through https://deeppbs.usc.edu.

For manuscripts utilizing custom algorithms or software that are central to the research but not yet described in published literature, software must be made available to editors and reviewers. We strongly encourage code deposition in a community repository (e.g. GitHub). See the Nature Portfolio guidelines for submitting code & software for further information.

Data

Policy information about availability of data

All manuscripts must include a data availability statement. This statement should provide the following information, where applicable:

- Accession codes, unique identifiers, or web links for publicly available datasets
- A description of any restrictions on data availability
- For clinical datasets or third party data, please ensure that the statement adheres to our policy

Datasets used for all analysis and associated custom scripts are deposited at https://doi.org/10.6084/m9.figshare.25678053 . Source data for figures and additional supplementary data are available. Accession codes for discussed structures from the Protein Data Bank: 2R5Y, 3Q05, 5X6G, 1L3L, 7CLI, 2R5Z, 1CIT, 1F4K, 1GJI, 1TC3, 2BSQ, 2C9L, 5ZGN, 1BBX, 1KLN, 1N5Y, 5YUZ, 1QAI, 1XC8, 6T8H, 4TUI, 1DH3, 7OH9, 1APL. UniProt accession codes for protein sequences discussed (folded with RFNA) Q8IUE0, Q6H878, O43680, Q4H376 . Accession codes for discussed experimental specificity data from JASPAR2022 and HOCOMOCOv11: MA1897.1, MA1568.1, MA1031.1, MA1572.1, MA0112.2, MA0112.3, ESR1_HUMAN.H11MO.0, NFKB2_HUMAN.H11MO.0.B. Mutagenesis experiment data used is available from SAMPDI website. MELD-DNA modeled complex data was taken from doi: 10.5281/zenodo.7501937.

Human research participants

Policy information about studies involving human research participants and Sex and Gender in Research.

Reporting on sex and gender	N/A
Population characteristics	N/A
Recruitment	N/A
Ethics oversight	N/A

Note that full information on the approval of the study protocol must also be provided in the manuscript.

Field-specific reporting

Please select the one below that is the best fit for your research. If you are not sure, read the appropriate sections before making your selection.

☒ Life sciences ☐ Behavioural & social sciences ☐ Ecological, evolutionary & environmental sciences

For a reference copy of the document with all sections, see nature.com/documents/nr-reporting-summary-flat.pdf

Life sciences study design

All studies must disclose on these points even when the disclosure is negative.

Sample size	We attempted to gather the largest, non-redundant dataset possible. All sample sizes for various datasets used in the study are provided through the manuscript and supplementary information.
Data exclusions	All filtering criteria were predetermined. Sample sizes were constrained by processing abilities of the various softwares used in the study. All descriptions for the same are provided through the manuscript and supplementary information.
Replication	Repeated model training and analysis were carried out at various intervals throughout the design of the study, fine tuning and through different stages of revision, leading to same conclusions.
Randomization	Sampling was involved in creation of cross validation dataset. For each sequence cluster, biological assemblies corresponding to upto 5 members were randomly sampled into one of five cross validation folds. Each model was trained on four folds and validated on the fifth fold. Biological assemblies not sampled into cross validation folds were resampled into a benchmark set.
Blinding	Standard five fold cross validation technique was used to train the models and a separate fully blindfold benchmark set was kept aside.

Reporting for specific materials, systems and methods

We require information from authors about some types of materials, experimental systems and methods used in many studies. Here, indicate whether each material, system or method listed is relevant to your study. If you are not sure if a list item applies to your research, read the appropriate section before selecting a response.

Materials & experimental systems

n/a	Involved in the study
☒	☐ Antibodies
☒	☐ Eukaryotic cell lines
☒	☐ Palaeontology and archaeology
☒	☐ Animals and other organisms
☒	☐ Clinical data
☒	☐ Dual use research of concern

Methods

n/a	Involved in the study
☒	☐ ChIP-seq
☒	☐ Flow cytometry
☒	☐ MRI-based neuroimaging
